# An indirect treatment comparison meta-analysis of digital versus face-to-face cognitive behavior therapy for headache

**DOI:** 10.1038/s41746-024-01264-9

**Published:** 2024-09-29

**Authors:** Yan-Bing Huang, Li Lin, Xin-Yu Li, Bo-Zhu Chen, Lu Yuan, Hui Zheng

**Affiliations:** The Acupuncture and Tuina School, Chengdu University of Traditional Chinese Medicine, Chengdu, China

**Keywords:** Headache, Disease prevention

## Abstract

Cognitive behavioral therapy (CBT) is effective for headache disorders. However, it is unclear whether the emerging digital CBT is noninferior to face-to-face CBT. An indirect treatment comparison (ITC) meta-analysis was conducted to assess the relative effects between them using standard mean differences (SMDs). Effective sample size (ESS) and required sample size (RSS) were calculated to demonstrate the robustness of the results. Our study found that digital CBT had a similar effect on headache frequency reduction (SMD, 0.12; 95%CI, −2.45 to 2.63) compared with face-to-face CBT. The ESS had 84 participants, while the RSS had 466 participants to achieve the same power as a non-inferior head-to-head trial. Digital CBT is as effective as face-to-face CBT in preventing headache disorders. Due to the heterogeneity (I^2^ = 94.5%, τ^2^ = 1.83) and the fact that most of the included studies were on migraine prevention, further head-to-head trials are warranted.

## Introduction

Headache disorders are among the most common and costly neurological disorders in the world^1^. Worldwide, 47% of the adult population suffers from active headache disorders, 10% from migraine, 38% from tension-type headache and 3% from chronic headache lasting more than 15 days per month^2^. Headache disorders not only cause disability, pain and loss of quality of life, as do other chronic diseases, but also have a higher societal cost. Indirect costs due to lost productivity, absenteeism or headache have also been reported to far exceed direct costs and increase with severity^3–5^. Therefore, headache prevention is essential.

Cognitive behavioral therapy (CBT), a non-pharmacological psychotherapy, has shown effectiveness in relieving psychiatric disorders, stress, chronic pain, and migraine^6–9^. CBT approaches integrate both behavioral and cognitive interventions. The underlying principle of CBT is that symptoms and dysfunctional behaviors are cognitively mediated. Therefore, improvement can be achieved by modifying dysfunctional thinking and beliefs^10^. Nevertheless, several obstacles impede the accessibility of face-to-face CBT. These include the high cost, the presence of multiple comorbidities that can be problematic for therapists and patients^9,11,12^, a paucity of trained providers with uneven geographic distribution, and even state licensure regulations prohibiting cross-border practice^13,14^. Thus, although effective, face-to-face CBT is not easy to implement in practice^9,11,12^.

Digital CBT is an emerging approach that provides support away from face-to-face clinical involvement^15^. Typical components of CBT are delivered through digital techniques, such as journaling, behavioral activation, exposure exercises, cognitive restructuring, relaxation, and a variety of other techniques and procedures^16^. The most intuitive difference between digital and face-to-face CBT is the method of delivery. Face-to-face CBT requires the patient and therapist to be simultaneously and fully engaged in the treatment process. The therapist must evaluate the patient’s beliefs, test them to see if they are accurate or not, and modify them according to reality^10^. The digital model, which depends on a confidential online platform, has been jointly developed by psychological professionals and programmers and has been applied to clinical treatments^17^. Depending on the specific digital forms and delivery details, digital CBT can be accompanied by therapist guidance^18^ or fully automated^19,20^. Definitely, the delivering contents are based on the principles of CBT^20,21^.

The advent of the Internet has made digital CBT more accessible. Digital CBT can address barriers such as therapist shortages through a culturally tailored digital format^22^. Digital CBT requires only a fraction of the therapist time required for face-to-face CBT^23^. This also means that the cost of digital CBT is subsequently reduced. When access to face-to-face CBT is limited for headache sufferers, highly accessible digital modalities may be considered by therapists. However, it is unclear whether emerging digital CBT therapies are non-inferior to face-to-face CBT, and we hypothesized that the two therapies would have similar effects on headache prevention. Therefore, the primary aim of this study is to compare the effectiveness of face-to-face with digital CBT based on the available evidence. The secondary aim is to estimate the effective sample size which produces the same precision and power as a non-inferior randomized controlled trial (RCT).

## Results

### Characteristics of the included RCTs

**Fig. 1:**
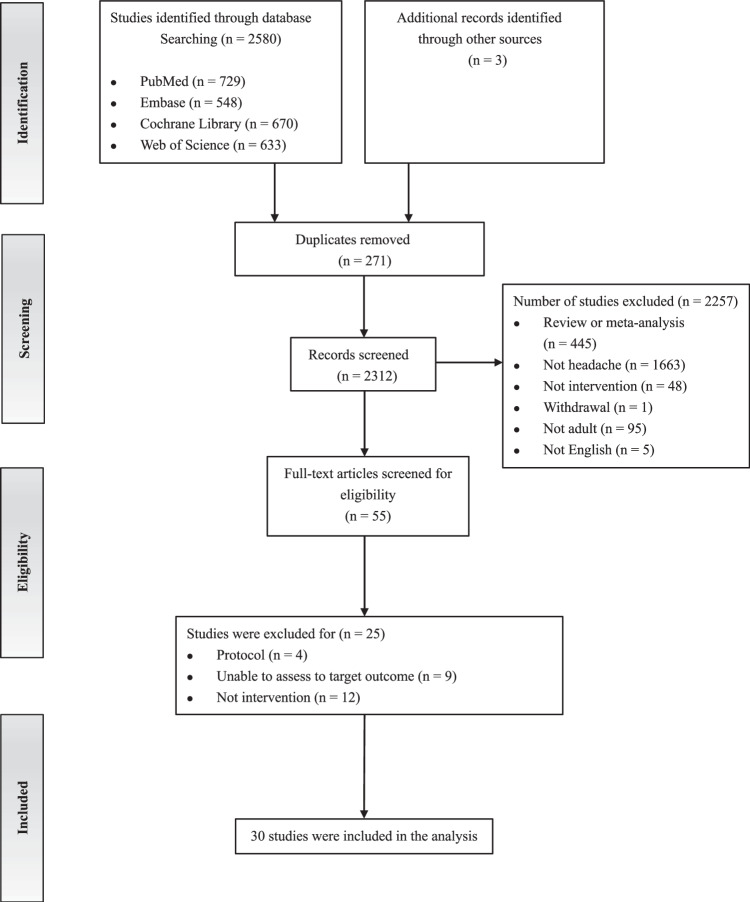
PRISMA flow diagram of literature search and study selection. The details of the literature search and study selection process have been shown. Boxes indicate the screening context of each step. Arrows indicate the order of screening as recommended by the PRISMA guidelines.

A total of 2583 studies were initially identified through database searches and additional sources. Titles and abstracts were screened, and 55 studies were selected for full-text review. Of these, 25 studies were excluded for not meeting our inclusion criteria. Ultimately, 30 studies were included in this analysis. Please refer to Fig. 1 for a detailed process.

Thirty studies were eligible, with 5 studies reporting digital CBT and 25 studies reporting face-to-face CBT. Three studies of digital intervention were self-help only, and two had therapist contact. Waiting-list, reported by 12 trials, was considered as a comparator for this analysis instead of sham CBT with limited studies. The study investigated five types of headaches: migraine, migraine combined with tension-type headache (TTH), chronic tension-type headache (CTTH), chronic post-traumatic headache (CPTH), and recurrent headache. A total of 2069 participants were included in the study, with a mean age of 40.26 years. The study population was drawn from North America (*n* = 11), Europe (*n* = 12), Australia (*n* = 4), the Middle East (*n* = 2), and Asia (*n* = 1). The majority of the studies had a higher proportion of female participants, with the exception of McGeary et al., which had 87% male participants. Of the 30 included studies, the International Classification of Headache Disorders (ICHD) criteria were used for all headache diagnoses. Treatment duration ranged from 6 to 26 weeks. Outcomes were measured by headache frequency as recorded in headache diaries. Table 1 shows the detailed characteristics of the included studies. Table 2 shows that no baseline or design differences were found between the two groups of studies. Adherence to face-to-face and digital CBT was 82.66% and 73.36%, respectively.

**Table 1:** Characteristics of the included RCT

Study	Country	Diagnostic criteria	Type of Headache	Intervention	Treatment duration (weeks)	Study duration (weeks)	Number of patients	Female (%)	Mean age	Outcomes
Digital Cognitive Behavior Therapy										
Hedborg^17^	Sweden	ICHD-II	Migraine	dCBT + massage vs. dCBT vs. TAU	24	44	76	69.88	47.73	Primary outcome: headache frequency
Kleiboer^18^	Netherlands	ICHD-II	Migraine	dCBT vs. waiting-list	8	40	368	85.33	43.60	Primary outcome: headache frequency
Andersson^19^	Sweden	ICHD-I	Recurrent headache	dCBT + telephone vs. dCBT	6	8	30	81.80	40.30	Primary outcome: headache days
Crawford^20^	America	ICHD-II	Migraine and Insomnia	dCBT vs. dCBT vs. dCBT	6	12	35	100.00	42.00	Secondary outcome: headache frequency
Ström^21^	Sweden	ICHD-I	Recurrent headache	dCBT vs. waiting-list	6	20	45	68.89	40.35	Primary outcome: headache days
Face-to-face Cognitive Behavior Therapy										
Kjeldgaard^47^	Danish	ICHD-II	CPTH	CBT vs. waiting-list	26	30	71	56.00	34.00	Primary outcome: headache frequency
McGeary^48^	America	ICHD-III	CPTH and Posttraumatic Stress Disorder	CBT vs. CPT vs. TAU	6	34	193	13.00	39.70	Secondary outcome: headache frequency
Martin^49^	Australia	ICHD-II	Migraine and/or TTH	CBT vs. TAU	12	30	43	72.24	40.64	Primary outcome: headache ratings
Martin^50^	Australia	ICHD-I	Migraine and TTH	CBT vs. Biofeedback vs. waiting-list	NA	48	50	72.55	44.00	Primary outcome: headache ratings
Soleimanian^51^	Iran	ICHD-III	Migraine	CBT vs. TAU	8	16	35	91.40	37.20	Primary outcome: headache days
Cousins^52^	England	ICHD-II	Migraine	CBT vs. TAU	5	16	53	82.20	39.00	Primary outcome: headache days
Calhoun^29^	America	ICHD- II	Transformed migraine	CBT vs. sham CBT	6	22	43	100.00	34.25	Secondary outcome: headache frequency
Smitherman^30^	America	ICHD-II	Migraine and comorbid insomnia	CBT vs. sham CBT	6	12	31	90.30	30.85	Primary outcome: headache frequency
Mansourishad^53^	Iran	ICHD-III	Migraine	CBT vs. waiting-list	4	16	26	100.00	32.15	Primary outcome: headache frequency
Wells^54^	America	ICHD-II	Migraine	CBT vs. TAU	8	12	19	89.47	45.55	Primary outcome: headache frequency
Wells^55^	America	ICHD-II	Migraine	CBT vs. TAU	8	36	76	92.13	43.90	Primary outcome: headache frequency
Seminowicz^56^	America	ICHD-III	Migraine	CBT vs. TAU	16	52	98	90.80	36.00	Primary outcome: headache days
Kiran^57^	India	ICHD-II	CTTH	CBT vs. TAU	8	16	50	78.00	32.06	Primary outcome: headache frequency
Day^58^	America	ICHD-II	Primary headache	CBT vs. waiting-list	8	9	34	88.90	41.70	Primary outcome: headache frequency
Simshäuser^59^	Germany	ICHD-III	Migraine	CBT vs. TAU	8	60	44	92.00	44.00	Primary outcome: headache days
Simshäuser^60^	Germany	ICHD-III	Migraine	CBT vs. waiting-list	8	28	48	88.90	45.25	Secondary outcome: headache frequency
Martin^61^	Australia	ICHD-III	Migraine and TTH	CBT vs. waiting-list	12	64	87	71.54	46.89	Primary outcome: headache frequency
Timo^62^	Germany	ICHD-III	Migraine	CBT vs. TAU vs. waiting-list	7	NA	106	89.62	46.60	Primary outcome: headache days
Seng^63^	America	ICHD-III	Migraine	CBT vs. waiting-list	8	16	60	90.70	40.10	Secondary outcome: headache days
Cathcart^64^	Australia	ICHD-II	CTTH	CBT vs. waiting-list	3	NA	41	61.90	45.52	Primary outcome: headache frequency
Grazzi^65^	Italy	ICHD-III	Migraine	CBT vs. TAU	12	48	24	NA	41.95	Primary outcome: headache days
Thorn^66^	America	ICHD-II	Migraine and TTH	CBT vs. CBT	NA	57	34	82.40	42.71	Primary outcome: headache frequency
Amy^67^	America	ICHD-II	Migraine and/or TTH	CBT vs. TAU	4	4	83	90.40	19.10	Secondary outcome: headache frequency
Empl^68^	Germany	ICHD-III	Migraine	CBT vs. waiting-list	6	6	51	91.84	42.60	Primary outcome: headache days
Fritsche^69^	Germany	ICHD-II	Migraine and MOH	CBT vs. TAU	5	48	115	90.67	48.05	Primary outcome: headache days

*NA* not available, *dCBT* digital Cognitive Behavior Therapy, *CTTH* ChronicTension-Type Headache, *TTH* Tension-Type Headache, *ICHD* the International Classification of Headache Disorders, *CPT* Cognitive Processing Therapy, *TAU* Treatment As Usual, *CPTH* Chronic Post-traumatic Headache.

**Table 2:** Characteristics of face-to-face and digital CBT studies as included in the analysis of headache

	Face-to-face studies	Digital studies	Face-to-face vs. Digital studies^a^
Number of samples (trials)	25	5	–
Mean number of patients (SD)	58.56 (4.90)	110.8 (12.32)	W = 73.5, *ρ* = 0.5588
Mean age (SD)	39.75 (10.87)	42.80 (11.10)	W = 76.0, *ρ* = 0.4694
Mean ratio of female patients (%)	82.00%	81.18%	W = 45.0, *ρ* = 0.4023
Mean durations of headache (years/SD)	18.57 (11.84)	13.16 (11.24)	W = 22.0, *ρ* = 0.2467
Mean treatment duration in weeks (SD)	8.58 (2.61)	10.00 (5.06)	W = 58.0, *ρ* = 0.9293
Adherence (%)	82.66%	73.36%	W = 37.0, *ρ* = 0.2302

^a^Based on two-sample Mann–Whitney-U test for continuous variables.

Detailed information on the risk of bias evaluations for each study can be found in Supplementary Information (see Supplementary Fig. 1). In summary, twelve trials were classified as having a low risk of bias, ten trials were identified as having some concerns regarding risk, and eight trials were deemed to have a high risk of bias.

### Headache frequency

**Fig. 2:**
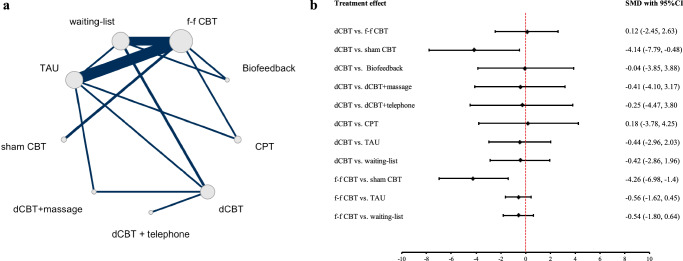
Network and pair-wise comparisons of indirect treatment comparison on headache frequency. **a** The network diagram illustrates the comparative analysis of the various measures. The size of the nodes is indicative of the sample size assigned to the various intervention groups. The thickness of the connecting lines between the different nodes indicates the number of studies included in the analysis. **b** Result of pair-wise comparisons of ITC meta-analysis on headache frequency. The standard mean differences (SMDs) and rhombs indicate the effect size values between the interventions. The error bars indicate the lower and upper limits of the 95% confidence interval (CI). The positioning of the rhombs to the left of the vertical line indicates that the intervention on the left is more effective than the intervention on the right. The inverse is also true.

Figure 2 shows the network and the comparison between face-to-face CBT and digital CBT for headache frequency. The study included 30 trials with a total of 2069 participants. The indirect treatment comparison analysis resulted in 11 pairwise comparisons, with evidence from both direct and indirect comparisons. Digital CBT (SMD 0.12; 95%CI, −2.45 to 2.63) had a similar therapeutic effect on headache frequency reduction compared with face-to-face CBT. Compared to waiting-list, digital CBT slightly improved headache frequency (SMD −0.42; 95%CI, −2.86 to 1.96). However, both face-to-face (SMD −4.26; 95%CI, −6.98 to −1.40) and digital CBT (SMD −4.14; 95%CI, −7.79 to −0.48) showed significantly better effects than sham CBT. The ranking plot (see Supplementary Table 1 and Supplementary Fig. 2) showed that digital CBT (SUCRA 0.61) is recommended after face-to-face CBT (SUCRA 0.68) and CPT (SUCRA 0.62). The analysis showed overall large heterogeneity (I^2^ = 94.5%, τ^2^ = 1.83). The CINeMA evidence was low or very low. The sensitive analysis pooling the studies reporting treatment as usual (TAU) or waiting-list was added (see Supplementary Table 2 and Supplementary Figure 3), which showed similar results to the main analysis.

### Subgroup analysis

**Fig. 3:**
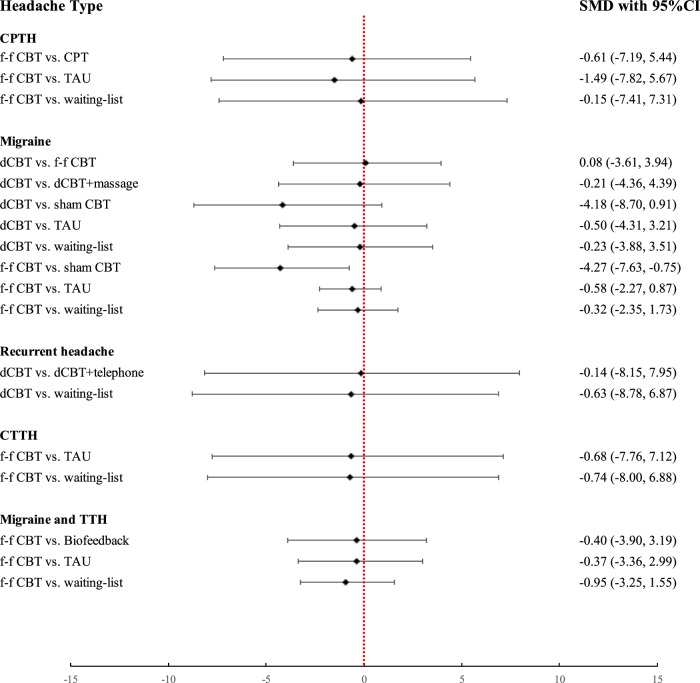
Subgroup analysis of headache frequency. The results of the subgroup analysis on the reduction of headache frequency are presented herewith. The standard mean differences (SMDs) and rhombs indicate the values of the effect size between the interventions. The error bars indicate the lower and upper limits of the 95% confidence interval (CI). The positioning of the rhombs to the left of the vertical line indicates that the intervention on the left is more effective, and vice versa.

Subgroup analyses of the primary outcome, which were prespecified in the registered protocol, were performed because of the wide range of headache disorders included. Figure 3 shows the subgroup analysis result of headache frequency, including CPTH, migraine, and migraine combined with TTH, CTTH, and recurrent headache. The comparison between digital CBT and face-to-face CBT was reported only for migraine. Compared with face-to-face CBT, digital CBT also showed similar efficacy in migraine prevention. Notably, the effect of face-to-face CBT was greater than that of sham CBT for migraine.

### The estimation of effective sample size

The effective sample size to achieve the same power and precision as a non-inferior head-to-head RCT with 466 participants was 84 participants for headache frequency reduction. IF = 18.03% indicated that accrual reached 18.03% of the required sample size (see Supplementary Table 3 for calculation details).

## Discussion

To the best of our knowledge, this meta-analysis is the first to compare the efficacy of face-to-face CBT and digital CBT for headache patients and to estimate the effective sample size. Based on the analysis of 30 studies with a total of 2069 participants, we found that digital CBT was similarly effective to face-to-face CBT in reducing headache frequency. Compared with waiting-list, face-to-face and digital CBT did not differ significantly in headache frequency reduction. However, both face-to-face CBT and digital CBT were superior to sham CBT in preventing headaches. However, only 2 studies with a total of 74 patients were included in the analysis of face-to-face versus sham CBT, and effective sample size calculation showed the insufficient number of studies and participants.

It is important to note that the results of our meta-analysis should be treated with caution. According to the Cochrane RoB2.0 assessment tool, more than half of the trials had some methodological problems. Only 12 out of 30 trials were considered to have a low risk of bias, and the CINeMA suggested that the level of evidence was low and very low. In addition, only one digital CBT trial had more than 100 participants, and the results indicated a large overall heterogeneity in the meta-analysis. Based on the subgroup analysis, it was found that the digital CBT data sources did not cover all types of headaches and the number of studies is limited. When the number of patients and events is sparse, pooled estimates of intervention effects tend to vary widely, resulting in a high risk of overestimation. Small sample sizes magnify treatment effects^24–27^.

Meanwhile, the indirect comparisons and subgroup analyses suggesting significant efficacy of digital and face-to-face CBT versus sham CBT in reducing migraine headache frequency should be viewed with caution. First, the small number of studies and sample sizes weakened the robustness of the available results. Second, the effect size of digital and face-to-face CBT versus sham CBT is larger than that of digital and face-to-face CBT versus TAU or waiting-list. This contradicts the previous findings of a study that examined the nonspecific effect of sham interventions. The results suggest that sham acupuncture and sham surgery have stronger non-specific effects than oral pharmacological placebos due to the high similarity of the interventions, blinding, and patient expectations^28^. The same sham CBT - “lifestyle modification” - was administered in both trials^29,30^. However, the mean change of sham CBT between baseline and treatment endpoint (6w) in terms of headache frequency was 0.7^29^ and −7.1^30^, respectively. Perhaps the differences in operational details, blinding, and participant expectations influenced the effect size. Thus, pooling the studies reporting sham CBT resulted in the lower non-specific effect of sham CBT pulling down another higher non-specific effect. Third, the TAU measures included treatment as usual, relaxation training, routine medical care, stress management, headache education, bibliotherapy, and progressive muscle relaxation and psychoeducation, which have a recognized therapeutic effect. Therefore, the pooled effect size of digital and face-to-face CBT versus sham CBT is not better than that of digital and face-to-face CBT versus TAU or waiting-list.

In addition, the presence or absence of therapist assistance (e.g., therapist guidance by phone^19^ or email^18^ to complete self-help^20^) also made the delivery of digital CBT inconsistent. One study^31^ suggested that the therapist’s manner of contact, such as a positive or reassuring attitude compared to a neutral attitude, may increase efficacy. Clinicians treating patients with migraine should be aware that a relevant part of the overall effect they observe in practice may be due to non-specific effects and that the size of such effects may differ between treatment modalities^28^. Our additional analyses showed that therapist-guided digital CBT was not significantly different from no therapist-guided digital CBT. And one trial showed that digital-only intervention plus telephone did not show a significant difference in improvements in clinical outcomes and dropout rates compared with self-help alone^19^. However, in a systematic review of major depression involving more than 11,000 participants, researchers found that additional human support, longer intervention time, and high adherence were associated with favorable treatment outcomes with digital CBT after adjusting for potential confounders^6^.

The effective sample size was calculated after accounting for heterogeneity according to Thorlund et al. ^32^. This may be a reasonable solution to heterogeneity and gives us an idea of the size of the study population with some power.

We also examined the efficacy of digital CBT beyond headache disorders. Despite the current low quality of evidence, digital CBT has shown some promise and improvement in pain catastrophizing and negative patient attitudes toward chronic low back pain^33^, gastrointestinal symptom-specific anxiety and disability in patients with refractory irritable bowel syndrome^34^, and tinnitus-related pain, distractibility, anxiety, and sleep disturbance in tinnitus^35^.

Although there is insufficient evidence to suggest that digital versus face-to-face CBT have similar efficacy for headache prevention, it was possible that patients may be willing to choose digital CBT given the benefits of digital CBT, such as cost-effectiveness^36^, time-individualized^20^ and culturally tailored^22^.

Currently, digital CBT is delivered primarily through websites or emails that provide specialized self-help materials or special web tools designed by professional counselors and web designers^17,19,21^. The website provides not only text, but also audio files, online presentations of self-directed exercises, and examples of common potential problems^19^. Other digital methods have also been used to treat headaches. In the Netherlands, for example, a large-scale ‘mass media’ treatment of headaches was attempted using a combination of educational television programs, written material and audio-recorded relaxation instructions^37^. In today’s world of advanced technology, the future clinical application of digital CBT is even more promising. Technologies such as virtual reality (VR) and artificial intelligence (AI) can simulate a real person delivering CBT to patients. This allows the program to guide and interact with the patient autonomously.

The heterogeneity in the results of our indirect treatment comparison can be attributed to several factors. First, although there were no differences in baseline means between the face-to-face and digital CBT groups, imbalances in baseline characteristics across trials and the statistical methods used to adjust for these characteristics may introduce heterogeneity into the analysis. While some studies use statistical models such as linear models to adjust for important baseline covariates, others use simpler statistics such as t-tests. Second, it is likely that the type of headache included in the analysis was the source of heterogeneity. The population, baseline headache severity and post-treatment headache relief were not balanced across headache subtypes.

Our study has limitations. First, our search may not have been comprehensive enough and some studies may have been missed. However, we had updated the search to some extent to ensure that it was comprehensive. Second, a small sample size would affect the stability of the results. However, we have at least identified the extent of the current lack of studies by calculating the effective sample size and the number of studies required. Third, despite the large number of headache subtypes, the number of correlated studies was insufficient. Many headache subtypes for which clinical evidence is urgently needed, such as chronic headache, were understudied. Our subgroup analyses could not be performed adequately and effectively. Finally, less than half of the studies were considered to be at low risk of bias, and the analysis lacked additional high-quality evidence.

Based on our indirect treatment comparisons, digital CBT is as effective as face-to-face CBT in preventing headache disorders. Given its wider reach or acceptability, digital CBT may be a viable alternative for headache prevention. However, adequately powered RCTs directly comparing digital and face-to-face CBT for headache are needed to validate these findings.

## Methods

### Registration

This network meta-analysis follows the guidelines set forth in the Preferred Reporting Items for Systematic Reviews and Meta-Analyses (PRISMA) statement^38^. It had been registered with the Open Science Framework (OSF) and assigned a digital object unique identifier (DOI) of Registration: 10.17605/OSF.IO/DU3T8.

### Ethical standard

Our study is an indirect treatment comparison meta-analysis using publicly available data from published trials, so no additional ethical approval is required.

### Eligibility criteria and study selection

Inclusion criteria were limited to RCTs with participants aged 18 years or older and diagnosed with headache according to the International Classification of Headache Disorders (ICHD). Relevant outcomes included headache frequency.

The exclusion criteria were as follows: (1) exclusion of duplicate articles; (2) exclusion of non-RCTs and open-label trials; (3) exclusion of duplicate published studies in the form of meeting abstracts, pooled analyses, or secondary analyses; (4) exclusion of other conditions not relevant to headache; (5) exclusion of study design protocols; (6) exclusion of incomplete studies; (7) exclusion of articles for which the full text was not available; (8) exclusion of non-English language articles; and (9) exclusion of therapeutic interventions not relevant to CBT.

Two evaluators screened the retrieved literature independently from the database search. A third evaluator checked the screening results and arbitrated when there was disagreement in study selection.

We updated the search period and eligibility criteria after discussion. It was determined that the open-label trials were qualified because of the specificity of CBT implementation and the difficulty of complete blinding.

### Search strategy

We conducted a comprehensive search of PubMed, Embase, Cochrane Controlled Register of Trials, and Web of Science databases from inception to April 15, 2024, with no language restrictions. The search strategy is described in the Supplementary Information (see Supplementary Table 4–7). In addition, we reviewed the reference lists of relevant reviews, meta-analyses, and network meta-analyses to identify potentially eligible studies.

### Risk of bias in individual studies

Risk of bias (ROB) was assessed independently by two authors using the ROB2.0 tool for assessing RCTs in the Cochrane Handbook^39^. The tool considers five domains: (i) random or concealed allocation sequence, (ii) deviations from the intended interventions, (iii) missing outcome data, (iv) measurement of the outcome, and (v) selection of the reported outcome. There are five response options for each domain: Yes (Y), Probably Yes (PY), Probably No (PN), No (N), and No Information (NI). The RCTs were classified into three levels based on their potential ROB: high risk, some concern, and low risk. This classification was used to assess the risk of bias in the evidence.

### Data extraction

Study data were collected by the reviewers using a predesigned data extraction form. The following items were extracted: author, year of publication, country, type of headache, diagnostic criteria for headache, intervention(s), duration of intervention(s), study period, sample size, mean age, proportion of female participants, and mean value from baseline to end of intervention of relevant outcome measures. Two reviewers were responsible for the extraction of study data, while a third reviewer was responsible for checking the accuracy and completeness of the extracted data. If any information was missing during the extraction process, the authors were contacted by e-mail to obtain additional data if possible.

### Outcome measurements

The primary outcome of the study was the frequency of headache, measured as the numbers or days of headache per month.

### Data analysis

Indirect treatment comparisons were conducted via Bayesian theory using Stan, a probabilistic programming language that implements full Bayesian statistical inference using Markov chain Monte Carlo techniques and penalized maximum likelihood estimation using Optimization^40^. We set N (0, 100^2) prior distributions for the treatment effects and study-specific intercepts and utilized half-N (5^2) prior for the heterogeneity standard deviation of the random-effect (RE) model. Baseline and study design differences between face-to-face and digital CBT were examined using the Mann-Whitney U test. The standard mean difference (SMD) and 95% confidence interval (CI) were calculated for headache frequency. A network diagram was used to demonstrate the comparisons between the different measures. The size of the nodes indicates the sample size assigned to the different intervention groups, while the thickness of the connecting lines between the different nodes corresponds to the number of studies. The study utilized forest plots and intervention ranking to compare the effectiveness of different interventions. Intervention rankings were determined using the surface under the cumulative ranking curve (SUCRA), which represents the percentage of effectiveness or safety of each treatment that would rank first compared with a hypothetical treatment with no uncertainty^41,42^. SUCRA values ranged from 0 to 1^41^. The proportion of variability due to heterogeneity was measured by the I^2^ statistic^43^ and the between-study variability was assessed by τ^2,^^44^. Mild heterogeneity can account for less than 30% of the variability, and substantial heterogeneity can account for well over 50%^43^. A τ^2^ estimate of approximately 0.04, 0.16, and 0.36 represented low, moderate, and high heterogeneity, respectively^45^. According to the pre-specified protocol, subgroup analyses of the primary outcome were performed based on headache type. All analyses were performed in R (version 4.4.1).

### The certainty of the evidence

The certainty of the evidence was assessed using the Confidence In Network Meta-Analysis (CINeMA). The level of evidence was summarized as high, moderate, low, or very low by the following six components: within-study bias, reporting bias, indirectness, imprecision, heterogeneity, and inconsistency^46^.

### The estimation of effective sample size

According to the study theory of Thorlund et al. ^32^, the effective sample size that would provide the same power and precision as a non-inferior RCT was estimated using the number of participants included in the indirect treatment comparison. If a non-inferior head-to-head randomized controlled trial (digital CBT versus face-to-face CBT) were to be designed with 1:1 randomization and a predicted difference of no more than 0.3, the required sample size was estimated. The information fraction (IF) measured how far we had come and how far we were from the required sample size. IF was the accrued number of patients (or statistical power), n, divided by the required sample size (or required statistical power)^32^.

## Supplementary information

Supplementary Information

References list

Code

## Data Availability

The datasets used and/or analyzed in the current study are available from the corresponding author upon reasonable request. The data supporting our study are presented in the article and in the supplementary material, and further questions can be directed to the corresponding author.

## Code availability

The underlying code for this study [and training/validation datasets] is not publicly available but may be made available to qualified researchers on reasonable request from the corresponding author.
