# Supplementary material for: An indirect treatment comparison meta-analysis of digital versus face-to-face cognitive behavior therapy for headache
Source: NPJ Digit Med. 2024 Sep 29;7:262. doi: 10.1038/s41746-024-01264-9 (PMC11439962; doi:10.1038/s41746-024-01264-9)
Supplement: Supplementary file 1 — Supplementary Information [file 41746_2024_1264_MOESM1_ESM.pdf]

Supplementary Information

Supplementary Table 1: The SUCRA value of CBT in headache frequency.

	p_rank [1]	p_rank [2]	p_rank [3]	p_rank [4]	p_rank [5]	p_rank [6]	p_rank [7]	p_rank [8]	p_rank [9]	SUCRA
d[f-f CBT]	0.07	0.26	0.51	0.72	0.89	0.97	1.00	1.00	1	0.68
d[Biofeedback]	0.21	0.36	0.45	0.52	0.60	0.69	0.82	0.98	1	0.58
d[CPT]	0.23	0.39	0.50	0.58	0.66	0.75	0.86	0.98	1	0.62
d[dCBT]	0.09	0.26	0.43	0.57	0.70	0.84	0.96	0.99	1	0.61
d[dCBT+telephone]	0.22	0.35	0.43	0.50	0.55	0.62	0.73	0.93	1	0.54
d[dCBT+massage]	0.16	0.27	0.36	0.44	0.52	0.62	0.75	0.96	1	0.51
d[sham CBT]	0.00	0.00	0.00	0.00	0.01	0.02	0.04	0.16	1	0.03
d[TAU]	0.01	0.05	0.15	0.31	0.52	0.73	0.92	1.00	1	0.46
d[waiting-list]	0.01	0.06	0.17	0.34	0.54	0.75	0.93	1.00	1	0.48

Supplementary Table 2. The SUCRA value of CBT in headache frequency after pooling the studies comparing the TAU and waiting list.

	p_rank[1]	p_rank[2]	p_rank[3]	p_rank[4]	p_rank[5]	p_rank[6]	p_rank[7]	p_rank[8]	SUCRA
d[f-f CBT]	0.07	0.28	0.55	0.80	0.94	0.99	1.00	1	0.66
d[Biofeedback]	0.19	0.36	0.48	0.58	0.67	0.81	0.98	1	0.58
d[CPT]	0.21	0.37	0.48	0.58	0.69	0.81	0.98	1	0.59
d[dCBT]	0.08	0.26	0.45	0.62	0.79	0.94	1.00	1	0.59
d[dCBT+telephone]	0.22	0.35	0.43	0.50	0.58	0.72	0.94	1	0.53
d[dCBT+massage]	0.21	0.36	0.46	0.55	0.63	0.80	0.97	1	0.57
d[sham CBT]	0.00	0.00	0.00	0.01	0.02	0.03	0.14	1	0.03
d[TAU/WL]	0.00	0.03	0.15	0.36	0.68	0.90	1.00	1	0.44

Footnote: f-f CBT: face-to-face Cognitive Behavior Therapy; dCBT: digital Cognitive Behavior Therapy; CPT: Cognitive Processing Therapy; TAU: Treatment As Usual; WL: waiting list.

Supplementary Table 3. Effective sample size calculation for headache frequency.

	**Waiting list as the comparator**
Trial count of dCBT versus f-f CBT	2:10
Number of patients of dCBT versus f-f CBT	413:487
I^2 of dCBT versus f-f CBT	62.7%:80.5%
The prior estimates of a treatment effect (d) of dCBT versus f-f CBT	0.12 [95%CI, -2.45 to 2.63]
the associated variance around that treatment effect of dCBT versus f-f CBT (V^2)	$V^2 = (SD)^2 = 1.3^2$
The effective number of trials	According to Table 1[1], the trial count ratio of headache frequency is 1:5 and the corresponding exact precision ratio is 7.2. With the trial count ratio of 1:5, we know that the required number of indirect comparison trials is 12 to achieve the same precision as 1 direct (head-to-head) trial, and 18 to achieve the same precision as 2 direct (head-to-head) trials. Now, the dCBT vs. f-f CBT study included 12 headache frequency trials. We achieved the required number of indirect comparison trials to produce the same precision as 1 direct (f-f CBT versus dCBT) trial. We included 12/18 indirect comparison trials to achieve the same precision as 2 direct (f-f CBT versus dCBT) trials. It should be noted, however, that Table 1[1] is only valid to the extent that sample sizes and population variances are similar across trials and to the extent that heterogeneity is absent or ignorable. In addition, each of these methods differs in simplicity and validity (the simpler being the least valid), so the authors have outlined the simplicity-validity trade-offs[1]. Therefore, the effective number of trials should be conservative.
The effective sample size	The more general formula for the effective indirect sample size without heterogeneity penalization is (as shown below):<br>$(n_{AC} \times n_{BC})/(n_{AC} + n_{BC})$<br>The more general formula for the heterogeneity-corrected effective sample size is (as shown below):<br>$[(n_{AC} \times (1 - I_{AC}{}^2)) \times (n_{BC} \times (1 - I_{BC}^2))]/[n_{AC} \times (1 - I_{AC}{}^2)) + (n_{BC} \times (1 - I_{BC}{}^2))]$<br>The comparison of dCBT and waiting list included 413 patients, with 62.7% heterogeneity. The comparison of f-f CBT and waiting list included 488 patients, but with 80.5% heterogeneity. Therefore, the effective indirect sample size without heterogeneity adjustment (n_{indirect}) and the heterogeneity-corrected effective sample size for headache frequency ($n_{\text{indirect-Pen-1}}$) are (as shown below):<br>$n_{\text{indirect}} = (413 \times 487)/(413 + 487) \approx 223$

	$n_{\text{indirect-Pen-1}} = [(413 \times (1-62.7\%)) \times (487 \times (1-80.5\%))]/[413 \times (1-62.7\%)) + (487 \times (1-80.5\%))] \approx 59$
	**TAU as the comparator**
Trial count of dCBT versus f-f CBT	1:12
Number of patients of dCBT versus f-f CBT	51:728
I^2 of dCBT versus f-f CBT	0.0%:93.3%
The prior estimates of a treatment effect (d) of dCBT versus f-f CBT	0.12 [95%CI, -2.45 to 2.63]
the associated variance around that treatment effect of dCBT versus f-f CBT (V^2)	V^2 = (SD)^2 = 1.3^2
The effective sample size	The more general formula for the effective indirect sample size without heterogeneity penalization is (as shown below): $$(n_{AC} \times n_{BC})/(n_{AC} + n_{BC})$$ The more general formula for the heterogeneity-corrected effective sample size is (as shown below): $$[(n_{AC} \times (1-I_{AC}{}^2)) \times (n_{BC} \times (1-I_{BC}^2))]/[n_{AC} \times (1-I_{AC}{}^2)) + (n_{BC} \times (1-I_{BC}{}^2))]$$ The comparison between dCBT and usual care included 51 patients, with 0.0% heterogeneity. The comparison between f-f CBT and usual care included 741 patients, also with 93.5% heterogeneity. Therefore, the effective indirect sample size without heterogeneity adjustment (n_{indirect}) and the heterogeneity-corrected effective sample size for headache frequency ($n_{\text{indirect-Pen-2}}$) are (as shown below): $n_{\text{indirect}} = (51 \times 728)/(51 + 728) \approx 48$ $n_{\text{indirect-Pen-2}} = [(51 \times (1-0.0\%)) \times (728 \times (1-93.3\%))]/[51 \times (1-0.0\%)) + (728 \times (1-93.3\%))] \approx 25$
the total effective sample size	And then the total effective sample size is the sum of the heterogeneity-corrected effective sample sizes calculated with different comparators. $n_{\text{total}} = n_{\text{indirect-Pen-1}} + n_{\text{indirect-Pen-2}} = 59 + 25 = 84$
Information Fraction (IF) for headache frequency	It is well established that the required sample size (RSS) for a head-to-head, non-inferiority RCT with 1:1 randomization[2]. The formula for the RSS for a non-inferior design is (as shown below): $$N = 2 \times 2 \times \left(\frac{z_{1-\alpha} + z_{1-\beta}}{\delta_0}\right)^2 \times s^2$$ N = required sample size; δ0= a clinically acceptable margin (small SMD = 0.3); S2= Polled standard deviation of both comparison groups[2]. We knew that

	the vaule of $z_{1-\alpha}$ and $z_{1-\beta}$ is 1.645 and 0.845 respectively based on the Z-value table.<br>$N = 2 \times 2 \times \left(\frac{1.645+0.845}{0.3}\right)^2 \times 1.3^2 \approx 466$<br>The information fraction (IF) is the accrued number of patients (or statistical information), n, divided by the required sample size (or required statistical information)[3,4]. This measure gives us an idea of how far we have come and how far we are from the benchmark—the required sample size[1].<br>IF = n/N<br>IF (headache frequency) = 84/466= 0.1803<br>The IF (headache frequency) indicates that the accrual of patients has reached 18.03 percent of the required sample size. In other words, if we need to conduct a large, non-inferiority randomized controlled trial of 466 people to compare the effect of headache frequency for face-to-face CBT versus digital CBT, this indirect treatment comparison of headache frequency represents 18.03 percent of the recruited population.

Footnote: The calculation process was based on the research theory authored of Thorlund et.al[1]. The comparator was determined from the network diagram generated by the indirect treatment comparison of intervention groups of interest. In this meta-analysis, the waiting list and treatment as usual (TAU) were common comparators for comparing face-to-face CBT (f-f CBT) with digital CBT (dCBT). The effective sample size (ESS) was calculated separately from the different meta-analyses of the different comparators (as shown in the above table).

Supplementary Table 4: Search strategy in PubMed.

#1	Headache
#2	Headaches or Head Pain or Head Pains or Pain, Head or Pains, Head or Cephalodynia or Cephalodynias or Cranial Pain or Cranial Pains or Pain, Cranial or Pains, Cranial or Cephalalgia or Cephalalgias or Cephalgia or Cephalgias or Generalized Headache or Generalized or Headaches or Headache, Generalized or Headaches, Generalized or Ocular Headache or Headache, Ocular or Headaches, Ocular or Ocular Headaches or Orthostatic Headache or Headache, Orthostatic or Headaches, Orthostatic or Orthostatic Headaches or Vertex Headache or Headache, Vertex or Headaches, Vertex or Vertex Headaches or Retro-Ocular Headache or Headache, Retro-Ocular or Headaches, Retro-Ocular or Retro Ocular Headache or Retro-Ocular Headaches or Sharp Headache or Headache, Sharp or Headaches, Sharp or Sharp Headaches or Throbbing Headache or Headache, Throbbing or Headaches, Throbbing or Throbbing Headaches or Unilateral Headache or Headache, Unilateral or Headaches, Unilateral or Unilateral Headaches or Hemicrania or Bilateral Headache or Bilateral Headaches or Headache, Bilateral or Headaches, Bilateral or Periorbital Headache or Headache, Periorbital or Headaches, Periorbital or Periorbital Headaches
#3	#1 or #2
#4	Cognitive Behavioral Therapy
#5	Behavioral Therapies, Cognitive or Behavioral Therapy, Cognitive or Cognitive Behavioral Therapies or Therapies, Cognitive Behavioral or Therapy, Cognitive Behavioral or Psychotherapy, Cognitive or Therapy, Cognitive or Cognitive Therapies or Therapies, Cognitive or Cognitive Therapy or Cognitive Behaviour Therapy or Behaviour Therapies, Cognitive or Behaviour Therapy, Cognitive or Cognitive Behaviour Therapies or Therapies, Cognitive Behaviour or Therapy, Cognitive Behaviour or Cognitive Psychotherapy or Cognitive Psychotherapies or Psychotherapies, Cognitive or Cognition Therapy or Cognition Therapies or Therapies, Cognition or Therapy, Cognitive Behavior or Behavior Therapies, Cognitive or Cognitive Behavior Therapies or Therapies, Cognitive Behavior or Therapy, Cognition or Behavior Therapy, Cognitive or Cognitive Behavior Therapy
#6	#4 or #5
#7	Randomized Controlled Trial
#8	Randomized Controlled Trial [Publication Type]
#9	Randomized Controlled Trials as Topic
#10	randomized controlled study or randomized controlled trial or randomized study or randomized trial or randomized placebo-controlled study or randomized placebo-controlled trial or randomized placebo controlled or randomized placebo-controlled or randomized double-blin* or randomized double blin* or randomized AND double-blin* or randomized AND placebo-controlled
#11	#7 or #8 or #9 or #10
#12	#3 and #6 and #11

Supplementary Table 5: Search strategy in Cochrane Library.

#1	Headache
#2	Headache, Throbbing or Throbbing Headache or Throbbing Headaches or Headaches, Throbbing or Headaches, Periorbital or Periorbital Headache or Periorbital Headaches or Headache, Periorbital or Bilateral Headaches or Bilateral Headache or Headache, Bilateral or Headaches, Bilateral or Orthostatic Headache or Orthostatic Headaches or Headache, Orthostatic or Headaches, Orthostatic or Retro Ocular Headache or Headaches, Retro-Ocular or Retro-Ocular Headache or Retro-Ocular Headaches or Headache, Retro-Ocular or Pains, Cranial or Headaches or Cephalgias or Cranial Pain or Cephalodynias or Cranial Pains or Cephalodynia or Cephalalgias or Pain, Cranial or Cephalgia or Cephalalgia or Head Pains or Head Pain or Pain, Head or Pains, Head or Unilateral Headache or Headaches, Unilateral or Hemicrania or Headache, Unilateral or Unilateral Headaches or Headache, Generalized or Headaches, Generalized or Generalized Headache or Generalized Headaches or Headache, Sharp or Sharp Headaches or Headaches, Sharp or Sharp Headache or Ocular Headache or Headache, Ocular or Headaches, Ocular or Ocular Headaches or Vertex Headache or Headaches, Vertex or Headache, Vertex or Vertex Headaches
#3	#1 or #2
#4	Cognitive Behavioral Therapy
#5	Cognition Therapies or Cognitive Behavior Therapies or Therapy, Cognitive Behavior or Behavior Therapy, Cognitive or Cognitive Psychotherapy or Behaviour Therapy, Cognitive or Psychotherapies, Cognitive or Therapies, Cognitive Behavioral or Therapy, Cognition or Cognitive Behavior Therapy or Cognitive Behaviour Therapies or Therapies, Cognitive or Cognition Therapy or Therapies, Cognition or Behaviour Therapies, Cognitive or Behavioral Therapies, Cognitive or Cognitive Therapy or Cognitive Behaviour Therapy or Therapy, Cognitive Behavioral or Therapy, Cognitive or Therapies, Cognitive Behavior or Psychotherapy, Cognitive or Therapy, Cognitive Behaviour or Cognitive Therapies or Behavior Therapies, Cognitive or Cognitive Behavioral Therapies or Behavioral Therapy, Cognitive or Therapies, Cognitive Behaviour or Cognitive Psychotherapies
#6	#4 or #5
#7	randomized controlled trials as topic
#8	Trials, Randomized Clinical or Clinical Trials, Randomized or Controlled Clinical Trials, Randomized
#9	#7 or #8
#10	#3 and #6 and #9

Supplementary Table 6: Search strategy in Web of Science.

#1	TS=(Headache) OR AB=(Headaches or Head Pain or Head Pains or Pain, Head or Pains, Head or Cephalodynia or Cephalodynias or Cranial Pain or Cranial Pains or Pain, Cranial or Pains, Cranial or Cephalalgia or Cephalalgias or Cephalgia or Cephalgias or Generalized Headache or Generalized or Headaches or Headache, Generalized or Headaches, Generalized or Ocular Headache or Headache, Ocular or Headaches, Ocular or Ocular Headaches or Orthostatic Headache or Headache, Orthostatic or Headaches, Orthostatic or Orthostatic Headaches or Vertex Headache or Headache, Vertex or Headaches, Vertex or Vertex Headaches or Retro-Ocular Headache or Headache, Retro-Ocular or Headaches, Retro-Ocular or Retro Ocular Headache or Retro-Ocular Headaches or Sharp Headache or Headache, Sharp or Headaches, Sharp or Sharp Headaches or Throbbing Headache or Headache, Throbbing or Headaches, Throbbing or Throbbing Headaches or Unilateral Headache or Headache, Unilateral or Headaches, Unilateral or Unilateral Headaches or Hemicrania or Bilateral Headache or Bilateral Headaches or Headache, Bilateral or Headaches, Bilateral or Periorbital Headache or Headache, Periorbital or Headaches, Periorbital or Periorbital Headaches)
#2	TS=(Cognitive Behavioral Therapy) OR AB=(Behavioral Therapies, Cognitive OR Behavioral Therapy, Cognitive OR Cognitive Behavioral Therapies OR Therapies, Cognitive Behavioral OR Therapy, Cognitive Behavioral OR Behavior Therapy, Cognitive OR Cognitive Behavior Therapy OR Cognitive Behaviour Therapy OR Behaviour Therapies, Cognitive OR Behaviour Therapy, Cognitive OR Cognitive Behaviour Therapies OR Therapies, Cognitive Behaviour OR Therapy, Cognitive Behaviour OR Cognitive Therapy OR Therapy, Cognitive Behavior OR Behavior Therapies, Cognitive OR Cognitive Behavior Therapies OR Therapies, Cognitive Behavior OR Cognitive Psychotherapy OR Cognitive Psychotherapies OR Psychotherapies, Cognitive OR Psychotherapy, Cognitive OR Therapy, Cognitive OR Cognitive Therapies OR Therapies, Cognitive OR Therapy, Cognition OR Cognition Therapy OR Cognition Therapies OR Therapies, Cognition)
#3	TS=(Randomized Controlled Trials as Topic) OR AB=(randomized controlled study OR randomized controlled trial OR randomized study OR randomized trial OR randomized placebo-controlled study OR randomized placebo-controlled trial OR randomized placebo controlled OR randomized placebo-controlled OR randomized double-blin* OR randomized double blin* OR randomized AND double-blin* OR randomized AND placebo-controlled)
#4	#1 and #2 and #3

Supplementary Table 7: Search strategy in Embase.

#1	(broad search) Headache
#2	(ab.ti) Headaches OR Head Pain OR Head Pains OR Pain, Head OR Pains, Head OR Cephalodynia OR Cephalodynias OR Cranial Pain OR Cranial Pains OR Pain, Cranial OR Pains, Cranial OR Cephalalgia OR Cephalalgias OR Cephalgia OR Cephalgias OR Generalized Headache OR Generalized OR Headaches OR Headache, Generalized OR Headaches, Generalized OR Ocular Headache OR Headache, Ocular OR Headaches, Ocular OR Ocular Headaches OR Orthostatic Headache OR Headache, Orthostatic OR Headaches, Orthostatic OR Orthostatic Headaches OR Vertex Headache OR Headache, Vertex OR Headaches, Vertex OR Vertex Headaches OR Retro-Ocular Headache OR Headache, Retro-Ocular OR Headaches, Retro-Ocular OR Retro Ocular Headache OR Retro-Ocular Headaches OR Sharp Headache OR Headache, Sharp OR Headaches, Sharp OR Sharp Headaches OR Throbbing Headache OR Headache, Throbbing OR Headaches, Throbbing OR Throbbing Headaches OR Unilateral Headache OR Headache, Unilateral OR Headaches, Unilateral OR Unilateral Headaches OR Hemicrania OR Bilateral Headache OR Bilateral Headaches OR Headache, Bilateral OR Headaches, Bilateral OR Periorbital Headache OR Headache, Periorbital OR Headaches, Periorbital OR Periorbital Headaches
#3	(combined) #1 or #2
#4	Cognitive Behavioral Therapy
#5	Behavioral Therapies, Cognitive OR Behavioral Therapy, Cognitive OR Cognitive Behavioral Therapies OR Therapies, Cognitive Behavioral OR Therapy, Cognitive Behavioral OR Behavior Therapy, Cognitive OR Cognitive Behavior Therapy OR Cognitive Behaviour Therapy OR Behaviour Therapies, Cognitive OR Behaviour Therapy, Cognitive OR Cognitive Behaviour Therapies OR Therapies, Cognitive Behaviour OR Therapy, Cognitive Behaviour OR Cognitive Therapy OR Therapy, Cognitive Behavior OR Behavior Therapies, Cognitive OR Cognitive Behavior Therapies OR Therapies, Cognitive Behavior OR Cognitive Psychotherapy OR Cognitive Psychotherapies OR Psychotherapies, Cognitive OR Psychotherapy, Cognitive OR Therapy, Cognitive OR Cognitive Therapies OR Therapies, Cognitive OR Therapy, Cognition OR Cognition Therapy OR Cognition Therapies OR Therapies, Cognition
#6	#4 or #5
#7	Randomized Controlled Trials as Topic
#8	randomized controlled study OR randomized controlled trial OR randomized study OR randomized trial OR randomized placebo-controlled study OR randomized placebo-controlled trial OR randomized placebo controlled OR randomized placebo-controlled OR randomized double-blin* OR randomized double blin* OR randomized AND double-blin* OR randomized AND placebo-controlled
#9	#7 or #8
#10	#3 and #6 and #9

Supplementary Table 8. PRISMA NMA Checklist of Items to Include When Reporting A Systematic Review Involving a Network Meta-analysis

Section/Topic	Item #	Checklist Item	Reported on Page #
**TITLE**			
Title	1	Identify the report as a systematic review *incorporating a network meta-analysis (or related form of meta-analysis).*	P1
**ABSTRACT**			
Structured summary	2	Provide a structured summary including, as applicable:<br>**Background:** main objectives<br>**Methods:** data sources; study eligibility criteria, participants, and interventions; study appraisal; and *synthesis methods, such as network meta-analysis.*<br>**Results:** number of studies and participants identified; summary estimates with corresponding confidence/credible intervals; *treatment rankings may also be discussed. Authors may choose to summarize pairwise comparisons against a chosen treatment included in their analyses for brevity.*<br>**Discussion/Conclusions:** limitations; conclusions and implications of findings.<br>**Other:** primary source of funding; systematic review registration number with registry name.	P2 (This section was completed according to the journal requirements)
**INTRODUCTION**			
Rationale	3	Describe the rationale for the review in the context of what is already known*, including mention of why a network meta-analysis has been conducted.*_	P3-4
Objectives	4	Provide an explicit statement of questions being addressed, with reference to participants, interventions, comparisons, outcomes, and study design (PICOS).	P4
**METHODS**			
Protocol and registration	5	Indicate whether a review protocol exists and if and where it can be accessed (e.g., Web address);	P11

		and, if available, provide registration information, including registration number.	
Eligibility criteria	6	Specify study characteristics (e.g., PICOS, length of follow-up) and report characteristics (e.g., years considered, language, publication status) used as criteria for eligibility, giving rationale. *Clearly describe eligible treatments included in the treatment network, and note whether any have been clustered or merged into the same node (with justification).*_	P11-12
Information sources	7	Describe all information sources (e.g., databases with dates of coverage, contact with study authors to identify additional studies) in the search and date last searched.	P12
Search	8	Present full electronic search strategy for at least one database, including any limits used, such that it could be repeated.	Supplementary Table 1-4
Study selection	9	State the process for selecting studies (i.e., screening, eligibility, included in systematic review, and, if applicable, included in the meta-analysis).	Figure 1
Data collection process	10	Describe method of data extraction from reports (e.g., piloted forms, independently, in duplicate) and any processes for obtaining and confirming data from investigators.	P13
Data items	11	List and define all variables for which data were sought (e.g., PICOS, funding sources) and any assumptions and simplifications made.	P13
**Geometry of the network**	**S1**	Describe methods used to explore the geometry of the treatment network under study and potential biases related to it. This should include how the evidence base has been graphically summarized for presentation, and what characteristics were compiled and used to describe the evidence base to readers.	P13-14
Risk of bias within individual studies	12	Describe methods used for assessing risk of bias of individual studies (including specification of whether this was done at the study or outcome level), and how this information is to be used in any data synthesis.	P12
Summary measures	13	State the principal summary measures (e.g., risk ratio, difference in means). *Also describe the use of additional summary measures assessed, such as treatment rankings and surface under the*	P13

		cumulative ranking curve (SUCRA) values, as well as modified approaches used to present summary findings from meta-analyses.	
Planned methods of analysis	14	Describe the methods of handling data and combining results of studies for each network meta-analysis. This should include, but not be limited to:<br>*Handling of multi-arm trials;*<br>*Selection of variance structure;*<br>*Selection of prior distributions in Bayesian analyses; and*<br>*Assessment of model fit.*	P13-14
**Assessment of Inconsistency**	**S2**	Describe the statistical methods used to evaluate the agreement of direct and indirect evidence in the treatment network(s) studied. Describe efforts taken to address its presence when found.	P14
Risk of bias across studies	15	Specify any assessment of risk of bias that may affect the cumulative evidence (e.g., publication bias, selective reporting within studies).	Supplementary Figure 1
Additional analyses	16	Describe methods of additional analyses if done, indicating which were pre-specified. This may include, but not be limited to, the following:<br>Sensitivity or subgroup analyses;<br>Meta-regression analyses;<br>*Alternative formulations of the treatment network; and*<br>*Use of alternative prior distributions for Bayesian analyses (if applicable).*_	P14-15
**RESULTS†**			
Study selection	17	Give numbers of studies screened, assessed for eligibility, and included in the review, with reasons for exclusions at each stage, ideally with a flow diagram.	P4
**Presentation of network structure**	**S3**	Provide a network graph of the included studies to enable visualization of the geometry of the treatment network.	Figure 2
**Summary of network geometry**	**S4**	Provide a brief overview of characteristics of the treatment network. This may include commentary on the abundance of trials and randomized patients for the different interventions and pairwise comparisons in the network, gaps of evidence in the treatment network, and potential biases	Figure 2

		reflected by the network structure.	
Study characteristics	18	For each study, present characteristics for which data were extracted (e.g., study size, PICOS, follow-up period) and provide the citations.	Table 1
Risk of bias within studies	19	Present data on risk of bias of each study and, if available, any outcome level assessment.	P5
Results of individual studies	20	For all outcomes considered (benefits or harms), present, for each study: 1) simple summary data for each intervention group, and 2) effect estimates and confidence intervals. *Modified approaches may be needed to deal with information from larger networks.*	None
Synthesis of results	21	Present results of each meta-analysis done, including confidence/credible intervals. *In larger networks, authors may focus on comparisons versus a particular comparator (e.g. placebo or standard care), with full findings presented in an appendix. League tables and forest plots may be considered to summarize pairwise comparisons.* If additional summary measures were explored (such as treatment rankings), these should also be presented.	P4-7
**Exploration for inconsistency**	**S5**	Describe results from investigations of inconsistency. This may include such information as measures of model fit to compare consistency and inconsistency models, *P* values from statistical tests, or summary of inconsistency estimates from different parts of the treatment network.	P6
Risk of bias across studies	22	Present results of any assessment of risk of bias across studies for the evidence base being studied.	P6
Results of additional analyses	23	Give results of additional analyses, if done (e.g., sensitivity or subgroup analyses, meta-regression analyses*, alternative network geometries studied, alternative choice of prior distributions for Bayesian analyses,* and so forth).	P6-7
**DISCUSSION**			
Summary of evidence	24	Summarize the main findings, including the strength of evidence for each main outcome; consider their relevance to key groups (e.g., healthcare providers, users, and policy-makers).	P7
Limitations	25	Discuss limitations at study and outcome level (e.g., risk of bias), and at review level (e.g.,	P10

		incomplete retrieval of identified research, reporting bias). *Comment on the validity of the assumptions, such as transitivity and consistency. Comment on any concerns regarding network geometry (e.g., avoidance of certain comparisons).*	
Conclusions	26	Provide a general interpretation of the results in the context of other evidence, and implications for future research.	P11 (This section was completed according to the journal requirements)
**FUNDING**			
Funding	27	Describe sources of funding for the systematic review and other support (e.g., supply of data); role of funders for the systematic review. This should also include information regarding whether funding has been received from manufacturers of treatments in the network and/or whether some of the authors are content experts with professional conflicts of interest that could affect use of treatments in the network.	P15 (This section was completed according to the journal requirements)

PICOS = population, intervention, comparators, outcomes, study design.

* Text in italics indicateS wording specific to reporting of network meta-analyses that has been added to guidance from the PRISMA statement.

† Authors may wish to plan for use of appendices to present all relevant information in full detail for items in this section.

Studies with intention-to-treat

Unique ID	Study ID	Experimental	Comparator	Outcome	Weight	Randomization process	Deviations from intended interventions	Missing outcome data	Measurement of the outcome	Selection of the reported result	Overall
Cousins 2015	Cousins 2015	real CBT	sham CBT	headache days	1	+	+	+	?	+	!
Calhoun 2006	Calhoun 2006	real CBT	sham CBT	frequency	1	?	+	+	?	+	!
Martin 2015	Martin 2015	real CBT	sham CBT	frequency	1	?	?	+	?	?	!
Martin 2007	Martin 2007	real CBT	sham CBT	frequency	1	?	+	+	-	?	-
Smitherman 2(	Smitherman 2016	real CBT	sham CBT	frequency	1	+	+	+	+	?	+
Soleimanian :	Soleimanian 202:	real CBT	sham CBT	headache days	1	?	+	+	+	+	+

+ Low risk
? Some concerns
- High risk

Studies with intention-to-treat

Unique ID	Study ID	Experimental	Comparator	Outcome	Weight	Randomization process	Deviations from intended interventions	Missing outcome data	Measurement of the outcome	Selection of the reported result	Overall
Hedborg 2011	Hedborg 2011	dCBT	control	heaadache freq	1	+	+	+	?	+	!
Andersson 20	Andersson 2003	ddCBT	dCBT+telephone	headache days	1	+	+	+	?	+	!
Kjeldgaard 2	Kjeldgaard 2014	f-f CBT	waiting list	headache frequ	1	+	+	+	-	+	-
McGeary 2022	McGeary 2022	f-f CBT	CPT	headache frequ	1	+	+	+	+	+	+
Ström 2000	Ström 2000	dCBT	sham CBT	headache days	1	+	+	+	+	+	+
Kleiboer 201	Kleiboer 2014	dCBT	waiting list	headache frequ	1	+	+	+	?	+	+
Fritsche 201	Fritsche 2010	face-to-face C	treatment as u	frequency freq	1	+	+	+	?	+	+
Day 2014	Day 2014	face-to-face C	waiting list	headache frequ	1	?	+	+	?	+	!
Simshäuser 2	Simshäuser 2022	face-to-face C	waiting list	headache frequ	1	+	+	+	+	+	+
Amy 2008	Amy 2008	face-to-face C	TAU	headache frequ	1	?	+	+	?	+	!
Wells 2014	Wells 2014	face-to-face C	TAU	headache frequ	1	+	+	+	?	+	+
Simshäuser 2	Simshäuser 2020	face-to-face C	TAU	headache frequ	1	+	+	-	+	?	-
Martin 2021	Martin 2021	face-to-face C	waiting list	headache frequ	1	+	+	+	+	+	+
Seminowicz 2	Seminowicz 2020	face-to-face C	TAU	headache frequ	1	+	+	+	+	+	+
Timo 2022	Timo 2022	face-to-face C	waiting list	headache frequ	1	+	+	+	-	+	-
Empl 2023	Empl 2023	face-to-face C	waiting list	headache frequ	1	+	+	+	?	+	!
Mansourishad	Mansourishad 20:	face-to-face C	waiting list	headache frequ	1	+	+	+	?	+	!
Wells 2021	Wells 2021	face-to-face C	TAU	headache frequ	1	+	+	+	+	+	+
Kiran 2014	Kiran 2014	face-to-face C	TAU	headache frequ	1	?	+	-	-	+	-
Seng 2019	Seng 2019	face-to-face C	waiting list	headache frequ	1	+	+	+	+	+	+
Crawford 202	Crawford 2020	digital CBT	digital CBT	headache frequ	1	+	+	+	-	+	-
Cathcart 201	Cathcart 2014	face-to-face C	waiting list	headache frequ	1	+	+	?	+	+	!
Grazzi 2019	Grazzi 2019	face-to-face C	TAU	headache frequ	1	?	-	+	-	+	-
Thorn 2007	Thorn 2007	face-to-face C	face-to-face C	headache frequ	1	+	+	-	-	+	-

+ Low risk
? Some concerns
- High risk

Supplementary Figure 1: The risk of bias of included RCTs. The graph displays the authors' judgments on risk of bias of each included study, presented as circles in three colors using the ROB2.0 tool. The red, yellow and green circles represent high, medium and low risk respectively.

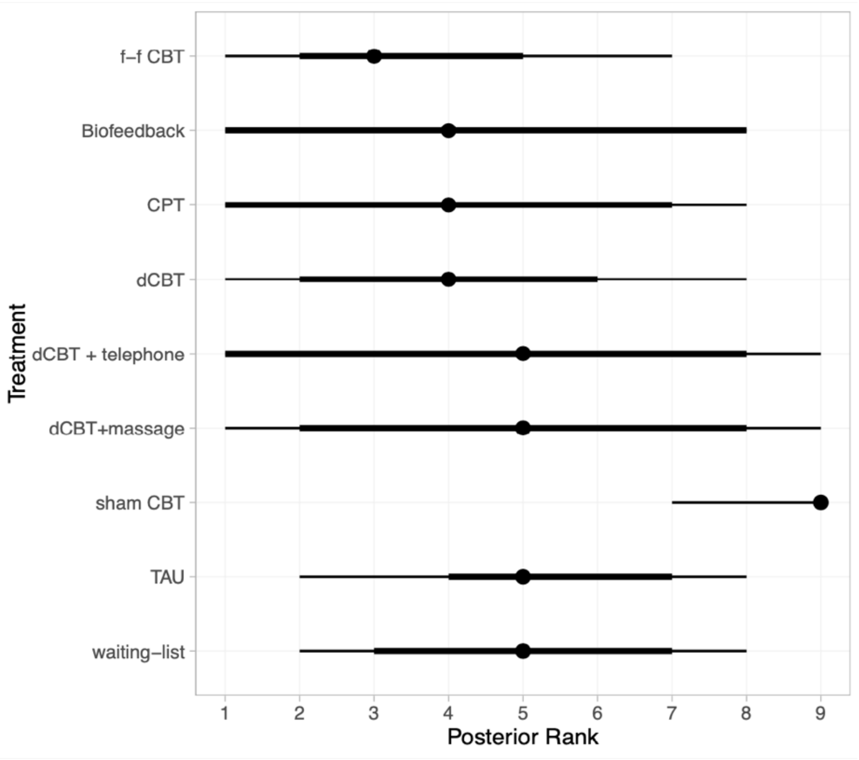

Supplementary Figure 2: The ranking chart of CBT on headache frequency. The graph indicates the ranking of CBT on headache frequency. The lower number indicates the better ranking.

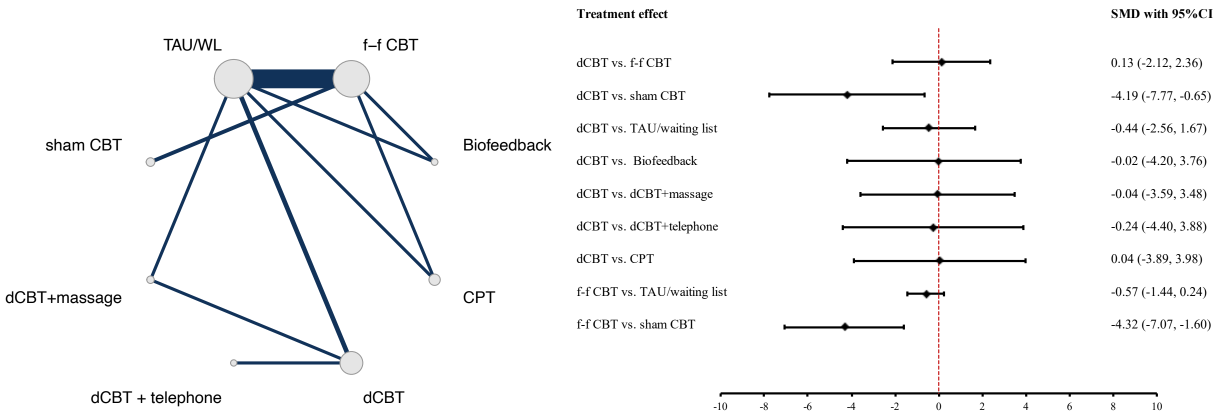

Supplementary Figure 3. Network and pair-wise comparisons of indirect treatment comparison on headache frequency after pooling the studies comparing the TAU and waiting list. The standard mean differences (SMDs) and rhombs indicate the values of the effect size between the interventions. The error bars indicate the lower and upper limits of the 95% confidence interval (CI). The positioning of the rhombs to the left of the vertical line indicates that the intervention on the left is more effective, and vice versa.

Supplementary References

1. Thorlund, K. & Mills, E. J. Sample size and power considerations in network meta-analysis. *Syst Rev* **1**, 41 (2012).

2. Zhong, B. How to calculate sample size in randomized controlled trial? *J Thorac Dis* **1**, 51–54 (2009).

3. Wetterslev, J., Thorlund, K., Brok, J. & Gluud, C. Trial sequential analysis may establish when firm evidence is reached in cumulative meta-analysis. *J Clin Epidemiol* **61**, 64–75 (2008).

4. Pogue, J. & Yusuf, S. Overcoming the limitations of current meta-analysis of randomised controlled trials. *Lancet* **351**, 47–52 (1998).
