# Supplementary material for: An indirect treatment comparison meta-analysis of digital versus face-to-face cognitive behavior therapy for headache
Source: NPJ Digit Med. 2024 Sep 29;7:262. doi: 10.1038/s41746-024-01264-9 (PMC11439962; doi:10.1038/s41746-024-01264-9)
Supplement: Supplementary file 2 — References list [file 41746_2024_1264_MOESM2_ESM.pdf]

References List

1. Jensen, R. & Stovner, L. J. Epidemiology and comorbidity of headache. *Lancet Neurol* **7**, 354–361 (2008).

2. Stovner, L. *et al.* The global burden of headache: a documentation of headache prevalence and disability worldwide. *Cephalalgia* **27**, 193–210 (2007).

3. Hu, X. H., Markson, L. E., Lipton, R. B., Stewart, W. F. & Berger, M. L. Burden of migraine in the United States: disability and economic costs. *Arch Intern Med* **159**, 813–818 (1999).

4. Berg, J. & Stovner, L. J. Cost of migraine and other headaches in Europe. *Eur J Neurol* **12 Suppl 1**, 59–62 (2005).

5. McCrone, P. *et al.* Service use and costs for people with headache: a UK primary care study. *J Headache Pain* **12**, 617–623 (2011).

6. Kambeitz-Ilankovic, L. *et al.* A systematic review of digital and face-to-face cognitive behavioral therapy for depression. *NPJ Digit Med* **5**, 144 (2022).

7. Anheyer, D. *et al.* Mindfulness-Based Stress Reduction for Treating Low Back Pain: A Systematic Review and Meta-analysis. *Ann Intern Med* **166**, 799–807 (2017).

8. Williams, A. C. de C., Eccleston, C. & Morley, S. Psychological therapies for the management of chronic pain (excluding headache) in adults. *Cochrane Database Syst Rev* **11**, CD007407 (2012).

9. Harris, P., Loveman, E., Clegg, A., Easton, S. & Berry, N. Systematic review of cognitive behavioural therapy for the management of headaches and migraines in adults. *Br J Pain* **9**, 213–224 (2015).

10. Knapp, P. & Beck, A. T. [Cognitive therapy: foundations, conceptual models, applications and research]. *Braz J Psychiatry* **30 Suppl 2**, s54-64 (2008).

11. Sullivan, A., Cousins, S. & Ridsdale, L. Psychological interventions for migraine: a

systematic review. *J Neurol* **263**, 2369–2377 (2016).

12. Harris, P., Loveman, E., Clegg, A., Easton, S. & Berry, N. Systematic review of cognitive behavioural therapy for the management of headaches and migraines in adults. *Br J Pain* **9**, 213–224 (2015).

13. Minen, M. T., Jalloh, A., Begasse de Dhaem, O. & Seng, E. K. Behavioral Therapy Preferences in People With Migraine. *Headache* **60**, 1093–1102 (2020).

14. Thomas, A. *et al.* Where are the Behavioral Sleep Medicine Providers and Where are They Needed? A Geographic Assessment. *Behav Sleep Med* **14**, 687–698 (2016).

15. Webb, C. & Orwig, J. Expanding our Reach: Telehealth and Licensure Implications for Psychologists. *J Clin Psychol Med Settings* **22**, 243–250 (2015).

16. Sullivan, A., Cousins, S. & Ridsdale, L. Psychological interventions for migraine: a systematic review. *J Neurol* **263**, 2369–2377 (2016).

17. Minen, M. T., Jalloh, A., Begasse de Dhaem, O. & Seng, E. K. Behavioral Therapy Preferences in People With Migraine. *Headache* **60**, 1093–1102 (2020).

18. Gratzer, D. & Khalid-Khan, F. Internet-delivered cognitive behavioural therapy in the treatment of psychiatric illness. *CMAJ* **188**, 263–272 (2016).

19. Andersson, G. & Carlbring, P. Internet-Assisted Cognitive Behavioral Therapy. *Psychiatr Clin North Am* **40**, 689–700 (2017).

20. Hedborg, K. & Muhr, C. Multimodal behavioral treatment of migraine: An Internet-administered, randomized, controlled trial. *UPSALA JOURNAL OF MEDICAL SCIENCES* **116**, 169–186 (2011).

21. Kleiboer, A., Sorbi, M., van Silfhout, M., Kooistra, L. & Passchier, J. Short-term

effectiveness of an online behavioral training in migraine self-management: a randomized controlled trial. *Behav Res Ther* **61**, 61–69 (2014).

22. Andersson, G., Lundström, P. & Ström, L. Internet-Based Treatment of Headache: Does Telephone Contact Add Anything? *Headache* **43**, 353–361 (2003).

23. Crawford, M. *et al.* Digital Cognitive Behavioral Therapy for Insomnia in Women With Chronic Migraines. *HEADACHE* **60**, 902–915 (2020).

24. Ström, L., Pettersson, R. & Andersson, G. A controlled trial of self-help treatment of recurrent headache conducted via the Internet. *J Consult Clin Psychol* **68**, 722–727 (2000).

25. Zhou, E. S. *et al.* Effect of Culturally Tailored, Internet-Delivered Cognitive Behavioral Therapy for Insomnia in Black Women: A Randomized Clinical Trial. *JAMA Psychiatry* **79**, 538–549 (2022).

26. Hedman, E., Ljótsson, B. & Lindefors, N. Cognitive behavior therapy via the Internet: a systematic review of applications, clinical efficacy and cost-effectiveness. *Expert Rev Pharmacoecon Outcomes Res* **12**, 745–764 (2012).

27. Thorlund, K. *et al.* Can trial sequential monitoring boundaries reduce spurious inferences from meta-analyses? *Int J Epidemiol* **38**, 276–286 (2009).

28. Trikalinos, T. A. *et al.* Effect sizes in cumulative meta-analyses of mental health randomized trials evolved over time. *J Clin Epidemiol* **57**, 1124–1130 (2004).

29. Ioannidis, J. & Lau, J. Evolution of treatment effects over time: empirical insight from recursive cumulative metaanalyses. *Proc Natl Acad Sci U S A* **98**, 831–836 (2001).

30. Gehr, B. T., Weiss, C. & Porzsolt, F. The fading of reported effectiveness. A meta-analysis of randomised controlled trials. *BMC Med Res Methodol* **6**, 25 (2006).

31. Meissner, K. *et al.* Differential effectiveness of placebo treatments: a systematic review of migraine prophylaxis. *JAMA Intern Med* **173**, 1941–1951 (2013).

32. Calhoun, A. H. & Ford, S. Behavioral sleep modification may revert transformed migraine to episodic migraine. *Headache* **47**, 1178–1183 (2007).

33. Smitherman, T. *et al.* Randomized Controlled Pilot Trial of Behavioral Insomnia Treatment for Chronic Migraine With Comorbid Insomnia. *HEADACHE* **56**, 276–291 (2016).

34. Di Blasi, Z., Harkness, E., Ernst, E., Georgiou, A. & Kleijnen, J. Influence of context effects on health outcomes: a systematic review. *Lancet* **357**, 757–762 (2001).

35. Thorlund, K. & Mills, E. J. Sample size and power considerations in network meta-analysis. *Syst Rev* **1**, 41 (2012).

36. Garg, S., Garg, D., Turin, T. C. & Chowdhury, M. F. U. Web-Based Interventions for Chronic Back Pain: A Systematic Review. *J Med Internet Res* **18**, e139 (2016).

37. Hanlon, I., Hewitt, C., Bell, K., Phillips, A. & Mikocka-Walus, A. Systematic review with meta-analysis: online psychological interventions for mental and physical health outcomes in gastrointestinal disorders including irritable bowel syndrome and inflammatory bowel disease. *Aliment Pharmacol Ther* **48**, 244–259 (2018).

38. Nyenhuis, N., Golm, D. & Kröner-Herwig, B. A systematic review and meta-analysis on the efficacy of self-help interventions in tinnitus. *Cogn Behav Ther* **42**, 159–169 (2013).

39. Haddock, C. K. *et al.* Home-based behavioral treatments for chronic benign headache: a meta-analysis of controlled trials. *Cephalalgia* **17**, 113–118 (1997).

40. de Bruijn-Kofman, A. T., van de Wiel, H., Groenman, N. H., Sorbi, M. J. & Klip, E. Effects of a mass media behavioral treatment for chronic headache: a pilot study. *Headache* **37**, 415–420

(1997).

41. Page, M. J. *et al.* The PRISMA 2020 statement: an updated guideline for reporting systematic reviews. *BMJ* **372**, n71 (2021).

42. Higgins, J. P. T. *et al.* The Cochrane Collaboration's tool for assessing risk of bias in randomised trials. *BMJ* **343**, d5928 (2011).

43. Carpenter, B. *et al.* Stan: A Probabilistic Programming Language. *J Stat Softw* **76**, 1 (2017).

44. Salanti, G., Ades, A. E. & Ioannidis, J. P. A. Graphical methods and numerical summaries for presenting results from multiple-treatment meta-analysis: an overview and tutorial. *J Clin Epidemiol* **64**, 163–171 (2011).

45. Veroniki, A. A., Straus, S. E., Rücker, G. & Tricco, A. C. Is providing uncertainty intervals in treatment ranking helpful in a network meta-analysis? *J Clin Epidemiol* **100**, 122–129 (2018).

46. Higgins, J. P. T. & Thompson, S. G. Quantifying heterogeneity in a meta-analysis. *Stat Med* **21**, 1539–1558 (2002).

47. Veroniki, A. A. *et al.* Methods to estimate the between-study variance and its uncertainty in meta-analysis. *Res Synth Methods* **7**, 55–79 (2016).

48. da Costa, B. R. & Juni, P. Systematic reviews and meta-analyses of randomized trials: principles and pitfalls. *Eur Heart J* **35**, 3336–3345 (2014).

49. Papakonstantinou, T., Nikolakopoulou, A., Higgins, J. P. T., Egger, M. & Salanti, G. CINeMA: Software for semiautomated assessment of the confidence in the results of network meta-analysis. *Campbell Syst Rev* **16**, e1080 (2020).

50. Kjeldgaard, D., Forchhammer, H., Teasdale, T. & Jensen, R. Cognitive behavioural treatment for the chronic post-traumatic headache patient: a randomized controlled trial. *JOURNAL*

OF HEADACHE AND PAIN **15**, (2014).

51. McGeary, D. *et al.* Cognitive Behavioral Therapy for Veterans With Comorbid Posttraumatic Headache and Posttraumatic Stress Disorder Symptoms A Randomized Clinical Trial. *JAMA NEUROLOGY* **79**, 746–757 (2022).

52. Martin, P. R. *et al.* Cognitive behavior therapy for comorbid migraine and/or tension-type headache and major depressive disorder: An exploratory randomized controlled trial. *Behav Res Ther* **73**, 8–18 (2015).

53. Martin, P. R., Forsyth, M. R. & Reece, J. Cognitive-behavioral therapy versus temporal pulse amplitude biofeedback training for recurrent headache. *Behav Ther* **38**, 350–363 (2007).

54. Soleimanian-Boroujeni, F., Badihian, N., Badihian, S., Shaygannejad, V. & Gorji, Y. The efficacy of transdiagnostic cognitive behavioral therapy on migraine headache: a pilot, feasibility study. *BMC Neurol* **22**, 230 (2022).

55. Cousins, S. *et al.* A pilot study of cognitive behavioural therapy and relaxation for migraine headache: a randomised controlled trial. *JOURNAL OF NEUROLOGY* **262**, 2764–2772 (2015).

56. Mansourishad, H., Togha, M., Borjali, A. & Karimi, R. Effectiveness of Mindfulness-Based Cognitive-Behavioral Therapy on Relieving Migraine Headaches. *Arch Neurosci* **In Press**, (2017).

57. Wells, R. E. *et al.* Meditation for migraines: a pilot randomized controlled trial. *Headache* **54**, 1484–1495 (2014).

58. Wells, R. *et al.* Effectiveness of Mindfulness Meditation vs Headache Education for Adults With Migraine: a Randomized Clinical Trial. *JAMA internal medicine* **181**, 317-328 (2021).

59. Seminowicz, D. A. *et al.* Enhanced mindfulness-based stress reduction in episodic migraine: a randomized clinical trial with magnetic resonance imaging outcomes. *Pain* **161**, 1837–1846

(2020).

60. Kiran, null, Girgla, K. K., Chalana, H. & Singh, H. Effect of rajyoga meditation on chronic tension headache. *Indian J Physiol Pharmacol* **58**, 157–161 (2014).

61. Day, M. A. *et al.* Mindfulness-based cognitive therapy for the treatment of headache pain: a pilot study. *Clin J Pain* **30**, 152–161 (2014).

62. Simshäuser, K., Lüking, M., Kaube, H., Schultz, C. & Schmidt, S. Is Mindfulness-Based Stress Reduction a Promising and Feasible Intervention for Patients Suffering from Migraine? A Randomized Controlled Pilot Trial. *Complement Med Res* **27**, 19–30 (2020).

63. Simshäuser, K. *et al.* Mindfulness-Based Cognitive Therapy as Migraine Intervention: a Randomized Waitlist Controlled Trial. *Int J Behav Med* **29**, 597–609 (2022).

64. Martin, P. R. *et al.* Integrating headache trigger management strategies into cognitive-behavioral therapy: A randomized controlled trial. *Health Psychol* **40**, 674–685 (2021).

65. T, K. *et al.* Efficacy of Cognitive-Behavioral Therapy for the Prophylaxis of Migraine in Adults: A Three-Armed Randomized Controlled Trial. *Frontiers in neurology* **13**, (2022).

66. Seng, E. *et al.* Does Mindfulness-Based Cognitive Therapy for Migraine Reduce Migraine-Related Disability in People with Episodic and Chronic Migraine? A Phase 2b Pilot Randomized Clinical Trial. *HEADACHE* **59**, 1448–1467 (2019).

67. Cathcart, S., Galatis, N., Immink, M., Proeve, M. & Petkov, J. Brief mindfulness-based therapy for chronic tension-type headache: a randomized controlled pilot study. *Behav Cogn Psychother* **42**, 1–15 (2014).

68. Grazzi, L. *et al.* ACT for migraine: effect of acceptance and commitment therapy (ACT) for high-frequency episodic migraine without aura: preliminary data of a phase-II, multicentric,

randomized, open-label study. *Neurol Sci* **40**, 191–192 (2019).

69. Thorn, B. *et al.* A randomized clinical trial of targeted cognitive behavioral treatment to reduce catastrophizing in chronic headache sufferers. *JOURNAL OF PAIN* **8**, 938–949 (2007).

70. Ab, W. & Ki, P. Migraines and meditation: does spirituality matter? *Journal of behavioral medicine* **31**, (2008).

71. Empl, M. *et al.* Effects of Introvision, a self-regulation method with a mindfulness-based perception technique in migraine prevention: a monocentric randomized waiting-list controlled study (IntroMig Study). *J Headache Pain* **24**, 146 (2023).

72. Fritsche, G. *et al.* Prevention of medication overuse in patients with migraine. *Pain* **151**, 404–413 (2010).

Supplementary References

1. Thorlund, K. & Mills, E. J. Sample size and power considerations in network meta-analysis. *Syst Rev* **1**, 41 (2012).
2. Zhong, B. How to calculate sample size in randomized controlled trial? *J Thorac Dis* **1**, 51–54 (2009).
3. Wetterslev, J., Thorlund, K., Brok, J. & Gluud, C. Trial sequential analysis may establish when firm evidence is reached in cumulative meta-analysis. *J Clin Epidemiol* **61**, 64–75 (2008).
4. Pogue, J. & Yusuf, S. Overcoming the limitations of current meta-analysis of randomised controlled trials. *Lancet* **351**, 47–52 (1998).
