# Supplementary material for: An indirect treatment comparison meta-analysis of digital versus face-to-face cognitive behavior therapy for headache
Source: NPJ Digit Med. 2024 Sep 29;7:262. doi: 10.1038/s41746-024-01264-9 (PMC11439962; doi:10.1038/s41746-024-01264-9)
Supplement: Supplementary file 3 — Code [file 41746_2024_1264_MOESM3_ESM.pdf]

Code.zip

File 1-Meta_R

```
install.packages("meta")
library(meta)


# calculating the standard mean difference and V^2
library(metafor)
data <- read.csv(file.choose())
data
dat <- escalc(m1i = mean.psyc,
              sd1i = sd.psyc,
              n1i = n.psyc,
              m2i = mean.cont,
              sd2i = sd.cont,
              n2i = n.cont,
              data = data,
              measure="SMD", digits=3)
dat

# between-studies comparison
data <- read.csv(file.choose())
data
m1 <- metacont(n.psyc, mean.psyc, sd.psyc, n.cont, mean.cont, sd.cont,
               data = data, sm = "SMD")
m1
```

File 2-Multinma_R

```
install.packages("ggplot2")
library(ggplot2)
install.packages("rstan")
install.packages("multinma")
options(mc.cores = parallel::detectCores())
data <- read.csv(file.choose())
data
df_net <- set_agd_arm(data,
                      study = studyn,
                      trt = trtn,
```

```
                                            y = diff,
                                            se = se_diff,
                                            sample_size = n)
df_net

#the network diagram
plot(df_net,
          weight_nodes = T,
          weight_edges = T,
          nudge = 0.2)+
    ggplot2::theme(legend.position = "bottom",
                              legend.box = "vertical",
                              text = element_text(size = 10))

# random effect model
df_fit_1 <- nma(df_net,
                            trt_effects = "random",
                            consistency="consistency")

#relative effect of pair-wise comparison of all interventions
df_1_releff_all <- relative_effects(df_fit_1,
                                                            all_contrasts = TRUE)
plot(df_1_releff_all, ref_line = 0)
print(df_1_releff_all)

#intervention ranking
df_1_ranks <- posterior_ranks(df_fit_1,
                                                lower_better=TRUE)
plot(df_1_ranks)

# cumulative ranking probability and SUCRA
df_1_cumrankprobs <- posterior_rank_probs(df_fit_1,
                                                                    sucra=TRUE,
                                                                    cumulative = TRUE)
plot(df_1_cumrankprobs)
df_1_cumrankprobs
```
